# The data paper: a mechanism to incentivize data publishing in biodiversity science

**DOI:** 10.1186/1471-2105-12-S15-S2

**Published:** 2011-12-15

**Authors:** Vishwas Chavan, Lyubomir Penev

**Affiliations:** 1 Global Biodiversity Information Facility Secretariat, Universitetsparken 15, DK 2100, Copenhagen, Denmark; 2 Institute of Biodiversity and Ecosystem Research, Bulgarian Academy of Sciences, and Pensoft Publishers, 13a Geo Milev Street, 1111 Sofia, Bulgaria

## Abstract

**Background**

Free and open access to primary biodiversity data is essential for informed decision-making to achieve conservation of biodiversity and sustainable development. However, primary biodiversity data are neither easily accessible nor discoverable. Among several impediments, one is a lack of incentives to data publishers for publishing of their data resources. One such mechanism currently lacking is recognition through conventional scholarly publication of enriched metadata, which should ensure rapid discovery of 'fit-for-use' biodiversity data resources.

**Discussion**

We review the state of the art of data discovery options and the mechanisms in place for incentivizing data publishers efforts towards easy, efficient and enhanced publishing, dissemination, sharing and re-use of biodiversity data. We propose the establishment of the 'biodiversity data paper' as one possible mechanism to offer scholarly recognition for efforts and investment by data publishers in authoring rich metadata and publishing them as citable academic papers. While detailing the benefits to data publishers, we describe the objectives, work flow and outcomes of the pilot project commissioned by the Global Biodiversity Information Facility in collaboration with scholarly publishers and pioneered by Pensoft Publishers through its journals *Zookeys*, *PhytoKeys*, *MycoKeys*, *BioRisk*, *NeoBiota*, *Nature Conservation* and the forthcoming *Biodiversity Data Journal*. We then debate further enhancements of the data paper beyond the pilot project and attempt to forecast the future uptake of data papers as an incentivization mechanism by the stakeholder communities.

**Conclusions**

We believe that in addition to recognition for those involved in the data publishing enterprise, data papers will also expedite publishing of fit-for-use biodiversity data resources. However, uptake and establishment of the data paper as a potential mechanism of scholarly recognition requires a high degree of commitment and investment by the cross-sectional stakeholder communities.

## Background

It is known that one of the effective strategies for addressing the growing biodiversity crisis is access to a range of biodiversity- and ecosystems-related data and information in a useful form. Furthermore, discovery of existing and prospective unpublished data needs to be encouraged, if our goal is to fill the extensive biodiversity knowledge gap that exists today. This emphasis on free and open access to biodiversity data is in tune with the call for open access to primary scientific data, which has been growing since 1991, beginning with Bromley Principles [1].

Since then, many statements, policies, and guidelines for open access to scientific data have appeared [2-23]. The Berlin Declaration of 2003 has been signed by 302 scientific bodies worldwide [18]. In 2004, the Organization for Economic Co-operation and Development (OECD) also recognized the importance of open access to primary scientific data [23]. Recently established initiatives such as Conservation Commons [24], the Global Earth Observation System of Systems (GEOSS) 10 year implementation plan [25], and the Intergovernmental Science-Policy Platform on Biodiversity and Ecosystem Services (IPBES) [26] recognized the importance of open access to primary scientific knowledge. Many scholarly publishers have joined in implementing the common principle that scientists must make their data available for independent use, without restrictions, once the data have been used in publications [27-34]. Recently, several of them emphasized the need for simultaneous publication of primary biodiversity data with scholarly publications and described some approaches to incorporate this practice in the routine publication process [35-38].

Editors of scientific journals can have an important role in promoting public deposition of scientific data [39]. However, these efforts are yet to yield any significant results because existing data remain unpublished, undiscovered and thus underused [40]. The majority of initiatives to make data accessible have focused on 'big science' rather than 'small science' [41]. We do not have a model for publication and discovery of data from small scale data authors, who collectively produce huge quantities of primary data, forming the so-called 'long tail' of science data [41,42].

Biodiversity research, as well biodiversity conservation and sustainable use, cannot be achieved if data are not preserved, discovered and made accessible [43]. Thus, discovery is a first step towards increased access to primary biodiversity data. However, our current progress in discovering biodiversity data resources emphasize the need for innovative mechanisms to speed up progress. We propose the establishment of the 'biodiversity data paper' as one possible mechanism to offer scholarly recognition through registration of priority, citability and dissemination of the efforts and investment by data publishers in authoring rich metadata. In context of this article, the term 'data publisher' is used in its widest sense. Data publishers include all data creators, data curators, data managers and data publishing networks/systems who form an integral part of data life cycle. Thus, data publishers are individuals, institutions or networks that facilitates discovery and access to primary biodiversity data through national, regional, thematic or global networks such as the Global Biodiversity Information Facility (GBIF). These are often also referred to as 'data providers' [44].

### Publishing and discovery of biodiversity data: the state of the art

Primary biodiversity data are the digital text or multimedia data records that detail the instance of an organism - the what, where, when, how and by whom of the organism's occurrence and recording [44,45]. Many the biodiversity data are neither accessible nor discoverable [46]. Currently the GBIF facilitates discovery of over 10,000 data resources, providing access to over 267 million primary biodiversity data records. However, this progress can be compared to scratching the surface of a huge iceberg. For instance, 6,500 natural history collections across the world are believed to be holding approximately 3 billion data records spanning the past 250 years of biodiversity research [47,48]. Ariño (2010) very conservatively estimated it to be 1.2 to 2.1 billion, of which only 3% is discoverable at the moment [49]. Although data from 'data-rich' nations are being discovered at a snail's pace, there are no definite efforts being made to ensure discovery of data resources from mega-biodiverse, developing and under-developed regions of the world. Most of the existing data discovery efforts are geared towards big projects or initiatives that constitute less that 20% of the estimated universe of biodiversity data: the remaining 80% of the data, not easily found by potential user, is called 'dark data' [50]. These include investigator-focused 'small data', locally generated 'invisible data' and 'incidental data', which are less well planned, poorly curated and unlikely to be visible to others. These dark data are in danger of being lost for want of an appropriate discovery mechanism [51]. According to Heidorn (2008), these dark data may be more important, because of their huge volume, than the data that can be easily discovered and used [50].

In summary, there is a lack of up-to-date, easy, fast, reliable and affordable discovery and access to a wide spectrum of primary biodiversity data. This leads to an unnecessary duplication of effort. Furthermore, verification of results become difficult and investment in research, data creation and collection remain under-realized as these data are currently trapped invisibly in institutional and individual cupboards, computers and disks. This is an obstacle to interdisciplinary and international research [46], as huge investment in data collection does not in any way ensure that the data are accessible now or that they will be accessible in future. Thus discovery of both digital and non-digital data resources is essential for ensuring access and enhanced use of biodiversity data.

### Publishing and discovery of biodiversity data: the constraints and challenges

The major reasons for this grim state of affairs are: (a) the lack of sustainable practices for data publishing; (b) the lack of easy-to-use tools and related guidelines for authoring metadata documents; (c) the difficulty of dealing with heterogeneity and diversity of standards, tools and numerous metadata extensions; (d) the cost of creation and maintenance of infrastructure by small- and medium-scale data publishers; and (e) the lack of professional reward structures or incentives. The first four of these causes are being addressed by various initiatives. The GBIF and its participants and standards bodies such as Biodiversity Informatics (also known as the Taxonomic Database Working Group, TDWG) are at various stages of development. However, the last cause, providing reward structure for professional recognition and/or incentives of other kinds, does not seem to have been addressed.

There is a lack of incentive for data publishers in authoring and publishing metadata. Because of the lack of acknowledgement for the extra work entailed, metadata are often poorly documented or, worse, not produced at all. Thus, adequate metadata are very much the exception and not the norm [52]. Generating even partial sets of metadata that conform to standards usually requires substantial amount of time and expertise [51]. This ghost of "what's in it for me?" is the root cause that prevents data publishers making concerted efforts to author enriched metadata and publish it [46]. Authoring metadata is definitely not considered to be original scientific effort. Data publishers will perhaps be prepared to provide enriched metadata, but it is still unusual to appreciate the necessary extra work for authoring, revising, updating and publishing metadata [53].

Providing and publishing enriched metadata might not look essential to data producers now, but the discovery of primary biodiversity data is essential and highly desirable to the general scientific effort [46]. Thus, without definite incentive mechanisms, the discovery of biodiversity data resources will continue to remain a dream, hampering our progress in the area of biodiversity science and nature conservation.

## The data paper

To overcome the impediment described above, we propose the biodiversity data paper as a mechanism to incentivize efforts and investment towards discovery and publishing of biodiversity data resources. We define a data paper as a scholarly publication of a searchable metadata document describing a particular online accessible dataset, or a group of datasets, published in accordance to the standard academic practices*.*

A data paper is a journal publication whose primary purpose is to describe data, rather than to report a research investigation. As such, it contains facts about data, not hypotheses and arguments in support of those hypotheses based on data, as found in a conventional research article. Its purposes are threefold: to provide a citable journal publication that brings scholarly credit to data publishers; to describe the data in a structured human-readable form; and to bring the existence of the data to the attention of the scholarly community.

The description should include several important elements (usually called metadata elements or 'description of data') that document, for example, how the dataset was collected, the taxa it covers, the spatial and temporal ranges and regional coverage of the data records, provenance information concerning who collected and who owns the data, details of which software was used to create the data or could be used to view the data, and so on (Table 1).

**Table 1:** GBIF Metadata Profile (GMP) implemented in the GBIF Integrated Publishing Toolkit for authoring metadata document

GBIF Metadata Profile (GMP) elements	Description
**abstract**	A brief overview describing the dataset.
additionalInfo	Any information that is not characterized by the other resource metadata fields.
additionalMetadata	A flexible field for including any other relevant metadata that pertains to the resource being described. This field allows EML to be extensible in that any XML-based metadata can be included in this element.
address	A container for multiple subfields that describe the physical or electronic address of the responsible party for a resource.
administrativeArea	The equivalent of a 'state' in the US or province in Canada. This field is intended to accommodate the many types of international administrative areas.
alternateIdentifier	This is the only identifier issued by the IPT for the metadata document; it is a persistent identifier.
associatedParty	A party associated with the resource. Parties have particular roles.
beginDate	A single time stamp signifying the beginning of some time period.
beginRange	The lower value in a range of numbers. Use to represent an exact number by omitting the 'endRange' value.
bibliography	A list of citations that form a bibliography on literature related to or used in the dataset.
boundingCoordinates	The four margins (N, S, E, W) of a bounding box, or when considered in latitude-longitude pairs, the corners of the box.
calendarDate	Used to express a date, giving the year, month and day. The format should be one that complies with ISO standard 8601. The recommended format for EML is YYYY-MM-DD, where Y is the four-digit year, M is the two-digit month code (01-12, where January = 01), and D is the two-digit day of the month (01-31). This field can also be used to enter just the year portion of a date.
characterEncoding	Contains the name of the character encoding. This is typically ASCII, UTF-8 or one of the other common encodings.
citation	A single citation for to use when citing the dataset.
city	Used for the city name of the contact associated with a particular resource.
collection	A container element for other elements associated with collections (for example collectionIdentifier, collectionName).
collectionIdentifier	The URI (LSID or URL) of the collection. In RDF, used as URI of the collection resource.
collectionName	Official name of the collection in the local language.
commonName	Applicable common names, which may be general descriptions of a group of organisms, if appropriate, for example invertebrates, waterfowl.
contact	Contains contact information for the dataset. This is the person or institution to contact with questions about the use, interpretation of a dataset.
country	Used for the name of the contact's country.
coverage	Describes the extent of the coverage of the resource in terms of its spatial, temporal and taxonomic extent.
creator	The person who created the resource (not necessarily the author of this metadata about the resource).
dataFormat	A container element for other elements that describe the internal physical characteristics of the data object.
dataset	A wrapper for all other elements relating to a single dataset.
deliveryPoint	Used for the physical address for postal communication, for example, GBIF Secretariat, Universitetsparken 15.
description	Contains general textual descriptions.
descriptor	Used to document domains (themes) of interest, such as climate, geology, soils or disturbances.
descriptorValue	Contains a general description, either thematic or geographic, of the study area.
designDescription	Contains general textual descriptions of research design. It can include detailed accounts of goals, motivations, theory, hypotheses, strategy, statistical design and actual work.
distribution	Provides information on how the resource is distributed. When used at the resource level, this element can provide only general information, but elements for describing connections to online systems are provided.
eastBoundingCoordinate	Defines the longitude of the eastern-most point of the bounding box that is being described.
electronicMailAddress	The email address for the party. It is intended to be an internet SMTP email address, which should consist of a username followed by the @ symbol followed by the email server domain name address.
endDate	A single time stamp signifying the end of some time period.
endRange	The upper value in a range of numbers.
externallyDefinedFormat	Information about a non-text or proprietary formatted object.
formatName	Name of the format of the data object, for example, ESRI Shapefile.
formatVersion	Version of the format of the data object.
formationPeriod	Text description of the time period during which the collection was assembled for example 'Victorian', '1922-1932' or 'c. 1750'.
funding	Used to provide information about funding sources for the project, such as grant and contract numbers or names and addresses of funding sources.
generalTaxonomicCoverage	A general description of the range of taxa addressed in the dataset or collection.
geographicCoverage	A container for spatial information about a resource; allows a bounding box for the overall coverage (in latitude and longitude), and also allows description of arbitrary polygons with exclusions.
geographicDescription	A short text description of a dataset's geographic areal domain. A text description is especially important to provide a geographic setting when the extent of the dataset cannot be well described by the 'boundingCoordinates'.
givenName	Can be used for first name of the individual associated with the resource, or for any other names that are not intended to be alphabetic, as appropriate.
hierarchyLevel	Dataset level to which the metadata applies; default value is 'dataset'.
individualName	Contains subfields so that a person's name can be broken down into parts.
intellectualRights	Contain a rights management statement for the resource, or a reference a service providing such information.
jgtiCuratorialUnit	A quantitative descriptor (number of specimens, samples or batches).
jgtiUnitRange	A range of numbers (x to x), with the lower value representing an exact number when the higher value is omitted.
jgtiUnitType	A general description of the unit of curation, for example, 'jar containing plankton sample'.
jgtiUnits	The exact number of units within the collection.
keyword	A keyword or key phrase that concisely describes the resource or is related to the resource. Each keyword field should contain one and only one keyword.
keywordSet	A wrapper element for the keyword and keywordThesaurus elements.
keywordThesaurus	The name of the official keyword thesaurus from which keyword was derived.
**language**	The language in which the resource (not the metadata document) is written.
livingTimePeriod	Time period during which biological material was alive (for paleontological collections).
metadata	Contains the additional metadata to be included in the document. This element should be used for extending EML to include metadata that is not already available in another part of the EML specification.
metadataLanguage	The language in which the metadata (as opposed to the resource being described by the metadata) is written.
**metadataProvider**	The party responsible for the creation of the metadata document.
methodStep	Allows for repeated sets of elements that document a series of procedures followed to produce a data object, including text descriptions of the procedures, relevant literature, software, instrumentation, source data and any quality control measures taken.
methods	Documents scientific methods used in the collection of this dataset. It includes information on items such as tools, instrument calibration and software.
northBoundingCoordinate	Defines the latitude of the northern-most point of the bounding box that is being described.
objectName	The name of the data object. This often is the filename of a file in a file system or that is accessible on the network.
online	Contains information for accessing the resource online represented as a URL connection.
onlineUrl	A link to associated online information, usually a website. When the party represents an organization, this is the URL to a website or other online information about the organization. If the party is an individual, it might be their personal website or other related online information about the party.
organisationName	The full name of the organization that is associated with the resource. This field is intended to describe which institution or overall organization is associated with the resource being described.
para	Allows for text blocks to be included in EML.
parentCollectionIdentifier	Identifier for the parent collection for this sub-collection. Enables a hierarchy of collections and sub-collections to be built.
personnel	Extends associatedParty with role information and is used to document people involved in a research project by providing contact information and their role in the project.
phone	Describes information about the responsible party's telephone (voice or fax) number.
physical	A container element for all of the elements that allow description of the internal/external characteristics and distribution of a data object (for example, dataObject, dataFormat, distribution).
positionName	Intended to be used instead of a particular person or full organization name. If the associated person who holds the role changes frequently, then positionName would be used for consistency; for example, GBIF Data Manager.
postalCode	Equivalent to a US zip code or the number used for routing to an address in other countries.
project	Contains information on the project in which the dataset was collected. It includes information such as project personnel, funding, study area, project design and related projects.
**pubDate**	The date on which the resource was published.
purpose	A description of the purpose of the resource/dataset.
qualityControl	Provides a location for the description of actions taken to either control or assess the quality of data resulting from the associated method step.
rangeOfDates	Intended to be used for describing a range of dates and/or times. It can be used multiple times to document multiple date ranges. It allows for two 'singleDateTime' fields, the first to be used as the beginning dateTime and the second to be used as the ending dateTime of the range.
resourceLogoUrl	URL of the logo associated with a resource.
role	Used to describe the role the party had with respect to the resource. Some potential roles include technician, reviewer and principal investigator.
sampling	Description of sampling procedures, including the geographic, temporal and taxonomic coverage of the study.
samplingDescription	Allows a text-based/human-readable description of the sampling procedures used in the research project. The content of this element would be similar to a description of sampling procedures found in the methods section of a journal article.
singleDateTime	Intended to describe a single date and time for an event.
southBoundingCoordinate	Defines the latitude of the southern-most point of the bounding box that is being described.
specimenPreservationMethod	Picklist keyword indicating the process or technique used to prevent physical deterioration of non-living collections. Expected to contain an instance from the Specimen Preservation Method Type Term vocabulary.
studyAreaDescription	Documents the physical area associated with the research project. It can include descriptions of the geographic, temporal and taxonomic coverage of the research location and descriptions of domains (themes) of interest, such as climate, geology, soils or disturbances.
studyExtent	Represents both a specific sampling area and the sampling frequency (temporal boundaries, frequency of occurrence). The geographic studyExtent is usually a surrogate (representative area of) for the larger area documented in 'studyAreaDescription'.
surName	Used for the last name of the individual associated with the resource. This is typically the family name of an individual, for example, the name by which s/he is referred to in citations.
taxonRankName	The name of the taxonomic rank for which the taxon rank value is provided, for example, phylum, class, genus, species.
taxonRankValue	The name representing the taxonomic rank of the taxon being described.
taxonomicClassification	Information about the range of taxa addressed in the dataset or collection.
taxonomicCoverage	A container for taxonomic information about a resource. It includes a list of species names (or higher level ranks) from one or more classification systems.
temporalCoverage	Specifies temporal coverage, and allows coverages to be a single point in time, multiple points in time, or a range of dates.
**title**	Provides a description of the resource that is being documented that is long enough to differentiate it from other similar resources. Multiple titles may be provided, particularly when trying to express the title in more than one language (use the 'xml:lang' attribute to indicate the language if not English).
url	The URL of the resource that is available online.
westBoundingCoordinate	Defines the longitude of the western-most point of the bounding box that is being described.

The definitions of the elements are taken from [64,85,86]. Mandatory elements when authoring metadata through IPT 2.0.2+ are in bold.

An important feature of data papers is that they should always be linked to the published datasets they describe, and that this link (a URL, ideally resolving a digital object identifier, doi) should be published within the paper itself. Conversely, the metadata describing the dataset held within data archives should include the bibliographic details, including a resolvable doi, of the data paper once that is published.

Many would argue that a data paper is by no means a new concept. The Ecological Society of America has published data papers in *Ecological Archives*[54] since 2000. *Earth System Science Data *[55], CMB data papers [56], *BMC Data Notes *[57] and the *International Journal of Robotics Research*[58,59] are a few sporadic instances of data publishers. However, a mainstream mechanism and associated software tools to generate data paper manuscripts from enriched metadata describing a data resource is still not in place.

Unique features of data publishers for biodiversity, as proposed here, include: (a) low technology and infrastructural overheads; (b) close links or interconnections with data publishing and scholarly publishing cycles; (c) an automated, 'push-button', conversion tool exporting metadata to a manuscript; and (d) minimal core metadata elements to reduce the time required for authoring the metadata document. As evident from the preceding discussion, the objective of the biodiversity data paper is to describe all types of biodiversity data resources, including environmental data resources. To show that a data paper is indeed an efficient mechanism for biodiversity data discovery, the GBIF, together with Pensoft Publishers, launched a pilot project to complete the whole cycle, from the GBIF metadata catalog, through peer review and editorial process, to the final scholarly publication in the form of a data paper. During the pilot phase, data publishers describing biodiversity data resources accessible through the GBIF network will be published in Pensoft's journals *Zookeys*, *PhytoKeys*, *MycoKeys*, *BioRisk*, *NeoBiota*, *Nature Conservation* and the forthcoming *Biodiversity Data Journal*. The respective data publishing policies and guidelines for authors and reviewers have recently been published on Pensoft's website [60] and widely circulated through the GBIF network and other related communications platforms [61].

### The GBIF Metadata Profile and Integrated Publishing Toolkit

Data papers for biodiversity, as envisaged by the pilot project, will use the GBIF Metadata Profile (GMP) to author the metadata document. The GMP was developed to standardize how biodiversity data resources are described through the GBIF network [62,63]. The GMP is primarily based on EML, the Ecological Metadata Language [64]. The GMP uses a subset of EML and extends it to include additional requirements. Table 1 lists the GMP elements and their descriptions.

This profile (GMP) can be transformed to other metadata formats, such as the International Standards Organization (ISO) 19139 metadata profile. In the GMP, there is a minimum set of mandatory elements required, but it is recommended that as many elements as possible be used to make the metadata as descriptive and complete as possible. There are various ways in which a metadata document conforming to GMP can be authored, such as using GBIF's Integrated Publishing Toolkit (IPT) metadata editor [65], the Darwin Core Spreadsheet template metadata form [66], or simply taking a metadata document and replacing fields of relevance with your own data. Once the metadata document is authored, it can be validated against the GMP schema. The GBIF IPT contains a user-friendly interface that makes authoring metadata easy. Once the user has inputted and saved the minimum required metadata, they can return to it at any time to add to or modify the metadata [63]. More information about the GBIF IPT can be found at [67].

The GBIF IPT makes it easy to share three types of biodiversity-related information: primary taxon occurrence data (also known as primary biodiversity data [44]), taxon checklists, and general metadata about data resources. An IPT instance and the data and metadata registered through the IPT is connected to GBIF Registry [68,69], indexed for publishing through the GBIF network and GBIF data portal [70], and made accessible for public use.

### The data paper: steps from metadata to manuscript

**Figure 1:**
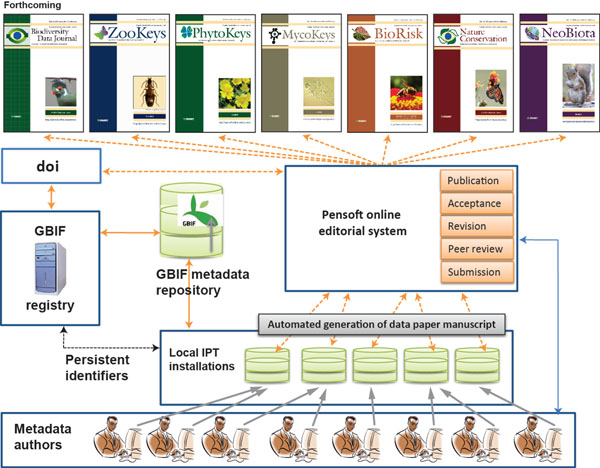
The GBIF/Pensoft workflow of data publishing and automated generation of data paper manuscripts.

As described in the previous section, data publishers will be able to author a metadata document by various means. However, to lower the technical barrier and make the process easy-to-adopt, an option of authoring metadata through IPT version 2.0.2 was developed. An added benefit of this option is that a conversion tool to automatically export metadata to a manuscript is embedded in IPT 2.0.2 at click of a button. As detailed in Table 2, this tool facilitates conversion of a metadata document into a traditional manuscript for submission to a journal. The step-by-step process in generation of a data paper manuscript from the metadata is depicted in Figure 1 and described below. A sample of a data paper manuscript is available [71].

**Table 2:** Structure of a data paper and its mapping to GBIF IPT Metadata Profile elements

Section/sub-section heading	Mapping with with GBIF IPT Metadata Profile elements
Title	Derived from the 'title' element. This is centred sentence without a full stop at the end.
Authors	Derived from the **'creator'**, **'metadataProvider'** and **'associatedParty'** elements. From these elements the combination of 'first name' and 'last name' are derived and separated by commas. Corresponding affiliations of the authors are denoted with superscript numbers (1, 2, 3,…) at the end of each last name. Centered.
Affiliations	Derived from the **'creator'**, **'metadataProvider'** and **'associatedParty'** elements. From these elements combinations of 'organization name', 'address', 'postal code', 'city', 'country' and 'email' constitute the address. If two or more authors share the same address, it is denoted by the same number.
Corresponding authors	Derived from the **'creator'** and **'metadataProvider'** elements. From these elements 'first name', 'last name' and 'email' are derived. Emails are written in parentheses. If there is more than one corresponding author, these are separated by commas. If creator and metadataProvider are the same, creator is reflected as corresponding author. Text is centered.
Received, Revised, Accepted and Published dates	These are to be manually inserted by the publisher of the data paper to indicate the dates of original manuscript submission, revised manuscript submission, acceptance of manuscript and publication of the manuscript as a data paper in the journal.
Citation	This is to be manually inserted by the publisher of the data paper. It is a combination of authors, year of data paper publication (in parentheses), title, journal name, volume, issue number (in parentheses), and doi of the data paper.
Abstract	Derived from the **'abstract'** element. Text is indented on the both sides.
Keywords	Derived from the **'keyword'** element. Keywords are separated by commas.
Introduction	
Taxonomic Coverage	Derived from the taxonomic coverage elements: '**taxonomicCoverage', 'taxonomicRankName'**, **'taxonomicRankValue'** and **'commonName'**. **'taxonomicRankName'** and **'taxonomicRankValue'** are derived together.
Spatial Coverage	Derived from the spatial coverage elements: **'geographicDescription'**, **'westBoundingCoordinate'**, **'eastBoundingCoordinate'**, **'northBoundingCoordinate'** and **'southBoundingCoordinate'**.
Temporal Coverage	Derived from the temporal coverage elements: **'beginDate'** and **'endDate'.**
Project Description	Derived from project elements: **'title'**, **'personnel'**, **'funding'**, **'studyAreaDescription'** and **'designDescription'**.
Natural Collections Description	Derived from project NCD elements: **'parentCollectionIdentifier'**, **'collectionName'**, **'collectionIdentifier'**, **formationPeriod'**, **'livingTimePeriod'**, **'specimenPreservationMethod'** and **'jgtiCuratorialUnit'**.
Methods	Derived from methods elements: **'methodStep'**, **'StudyExtent'**, **'samplingDescription'** and **qualityControl'**.
Dataset Descriptions	Derived from physical and other elements: **'objectName'**, **'characterEncoding'**, **'formatName'**, **'formatVersion'**, **'online/URL'**, **'pubDate'**, **'language'** and **'intellectualRights'**.
Additional Information	Derived from the **'additionalInfo'** element.
References	Derived from the **'citation'** element.

1. The data publisher completes the metadata for a biodiversity resource dataset using the metadata editor in IPT 2.0.2 or later versions. IPT assigns a persistent identifier to the authored metadata. A list of IPT installations supporting authoring of the data paper is accessible at [72].

2. Once the metadata are completed to the best of the author's ability, a data paper manuscript can be generated automatically from these metadata using the automated tool available within IPT 2.0.2+ (menu: Manage Resources - RTF download).

3. The author checks the created manuscript and then submits it for publication in the data paper section through the online submission system of an appropriate Pensoft journal (*Zookeys*, *PhytoKeys*, *MycoKeys*, *BioRisk*, *NeoBiota*, *Nature Conservation* or the forthcoming *Biodiversity Data Journal*).

4. The manuscript undergoes peer review according to the journal's policies and the guidelines for reviewers of data papers [60]. After review, and in the event of acceptance, the manuscript is returned to the authors by the editor alongside the reviewers' and editorial comments, for any required pre-publication modifications.

5. The corresponding author inserts all accepted corrections or additions recommended by the reviewers and the editor in the metadata, thereby improving the metadata document itself. Once the metadata document has been improved, it is made available on the IPT 2.0.2+ by pressing the Publish button in the Manage Resources menu (menu: Manage Resources - RTF download).

6. The final revised version of the manuscript is then created using the same automated metadata-to-manuscript conversion tool within IPT 2.0.2+ (menu: Manage Resources - RTF download) as was used to create the initially submitted draft.

7. Once the manuscript is accepted, it goes to a proofing stage, at which point submission, revision, acceptance and publication dates are added and a doi is assigned to the data paper. This facilitates persistent accessibility of the online scholarly publication.

8. Once the final proofs are approved, the data paper is published in four different formats: (a) print format; (b) PDF format, identical to the print version; (c) semantically enhanced HTML to provide internal cross-linking between sections, citations, references and links to external resources, and (d) final published XML to be archived in PubMed Central and other archives to facilitate future data mining.

9. After publication, the doi of the data paper is linked with the persistent identifier of the metadata document registered in the GBIF Registry [68], which is given in the data paper. This provides multiple cross-linking between the data resource, its corresponding metadata and the corresponding data paper.

10. Depending on the journal's policies and scope, the published data paper will be actively disseminated through the world's leading indexers and archives, such as Web of Knowledge (Thomson Reuters), PubMed Central, Scopus, Zoological Record, Google Scholar, CAB Abstracts, Directory of Open Access Journals (DOAJ), EBSCO and others.

Through the commissioning of data publishers as described above, close links will eventually be established between some advanced journal review systems (for example, open review systems and/or customized review system) and data publishing and discovery infrastructure (especially metadata catalogs). The data paper, being a peer-reviewed scholarly publication, can be recorded in citation indexes; it can therefore be used as performance evaluation mechanism.

### The data paper: peer review

Peer review of the potential data paper manuscript is expected to evaluate completeness and quality of the metadata. This may include the validity of methods used and standards conformance during the collection, management and curation of data. To meet the reviewers' expectations for accuracy and usefulness, the metadata needs to be as complete and descriptive as possible. This might require a review of the dataset itself. Depending on the journal's business model and policies, several types of review patterns or methods can be adopted. These include pre-acceptance review, open review and/or post-publication review. Pensoft's journals have adopted conventional pre-publication review as a routine method to enhance the completeness, reliability and accuracy of the metadata, thereby improving the use and relevance of the data resource. In the future, an open peer review system will be implemented through the *Biodiversity Data Journal*, currently established by Pensoft Publishers within the ViBRANT project [73].

## Discussion

In this section we shall discuss three key issues: benefits, further enhancements and mainstreaming of data publishers.

### The data paper: benefits

We believe that, if implemented in letter and spirit, data papers will address the issue of acknowledgement, an incentive to data publishers for their efforts in authoring rich metadata of a resource dataset. Data publishers will be credited through: (a) registering of priority and authorship in a conventional scholarly publication in any suitable journal; (b) indexing and citation of data publications in the same way as every research paper, which brings benefits to authors in recognition and career building; (c) the ability to trace usage and citations of published data; and (d) metadata published as a data paper being stored and archived in various ways, providing a persistent description of the corresponding data resource over time [35,74]. Furthermore, the data paper enables a division of labor in which those possessing the resources and skills can perform the experiments and observations needed to collect potentially interesting datasets, and manage, curate, discover and publish these datasets, so that many parties, each with a unique background and ability to analyze the data, can make use of them as they see fit [75].

Data produced are collected at the expense of the efforts of people and institutions, and usually funded by society, and so should be published, cited, used and re-used, separately or collated with other data. Data will be rendered, indexed, discoverable, browsable and searchable through the GBIF infrastructure. Data can be integrated through GBIF's infrastructure with other datasets across space, time and taxonomic groups, bringing recognition and new possibilities for collaboration to the authors. Datasets, metadata and respective data publishers are inter-linked to expedite and mutually extend the dissemination, for the benefit of the authors and society.

Increased and straightforward discovery of data resources would prevent duplication of effort in collecting data, for example from the same areas at the same time by different research groups. By contrast, it would open a window of collaboration between research groups and between data publishers. Discovery of data resources will also prevent potential misuse, as it will bring clarity with regard to ownership and custodianship of the data. In fact, efficient discovery of data resources will always bring advantages to researchers and data publishers.

Enrichment of metadata documents describing fitness for use of data resources will increase the usability, verifiability and credibility of those resources. Because data publishers will provide recognition to those involved in the management, discovery and publishing of biodiversity data, data resources locked in institutional and individual closets are likely to be discovered earlier than later. An early uptake of the data paper mechanism by the data publishers in data-rich and/or biodiversity-rich regions will result in greater uniformity of biodiversity data discovery and accessibility in the near future. For legacy data resources, such as natural history collections, data publishers will pave the way towards demand-driven digitization and publishing [76,77]. Furthermore, data papers could be a step towards long-term archiving and publishing of data resources.

### The data paper: further enhancements

Persistent identifiers are codes that are effectively permanently assigned to certain objects; each distinct persistent identifier can be defined as "a unique identification code that is applied to 'something', so that the 'something' can be unambiguously and permanently referenced" [78]. Persistent identifiers are essential for data papers. In addition to metadata, and its corresponding data paper, persistent identifiers of datasets could be assigned to facilitate deep data citation [46]. Allocation of persistent identifiers to data publishers and to an individual datum, and also versioning, should be explored. The ability to assign and resolve heterogeneous persistent identifiers for a data resource, its metadata and the data papers associated with it needs to be implemented [46].

There is a need for a controlled vocabulary to make the metadata authoring process straightforward and to enhance the quality and usability of the authored metadata document. A data paper needs to be an integral part of the data management process. Therefore, a data paper as conceptualized by us is based solely on metadata. However, the content of a data paper can further be enhanced with interpretive analysis of the data being described through metadata. These could include taxonomic, geospatial or temporal assessment of data and its potential of integration with other types of data resources. A data paper including a taxonomic checklist and/or the data themselves could be other possible enhancements.

An additional and potentially huge resource for the publication and discovery of primary biodiversity data is the Darwin Core Archive (DwC-A) format [79]. This format includes a set of text files in a tabular format, such as a comma-separated or tab-separated list, with a simple descriptor file to inform others how the data are organized. The format is defined in the Darwin Core text guidelines [80]. Darwin Core is no longer strictly bound to species occurrence data (primary biodiversity data), and together with the Dublin Core [81] (on which its ideas are based), it is used by GBIF and others to encode data about organism names, taxonomies, taxon checklists and species information. The DwC-A format is available and can be exported through IPT; it can be saved as a separate data package and could be collated with other data published in the same format. The Darwin Core Archive can also be published as a data package supplementary to a particular taxonomic revision or checklist. Thus, the DwC-A data underlying a taxonomic paper will be cross-linked between the source of publication and the GBIF metadata catalog. Recently, the use of DwC-A for publishing occurrence data has been pioneered by *ZooKeys *[82].

Data papers will be useful only if they can be linked with the data in real time without any further requirements, effort or barriers for data users. This calls for data publishers to be closely linked with data archival system or data publishing processes. We believe that data papers will drive the long-term archival of data and the persistent publication of data resources through one or more access points. The success of data papers as a mechanism for data discovery closely linked with deep data citation practices will acknowledge the efforts of all actors involved in data creation, data management and data publishing process.

As evident from this discussion, there are many people who should take pivotal roles in mainstreaming the data paper. However, the potential role of academic and scholarly publishers is crucial for the success of the data paper as a mechanism of discovery, sharing, collation, use and re-use of biodiversity data.

### The data paper: how to mainstream?

Mainstreaming of the data paper concept calls for cultural change and socio-political support, commitment and collaborations from all key stakeholders in biodiversity research and conservation. Publishing data as a mandatory requirement in research project proposals, subsequent grants and individual performance assessment is essential and seems to be becoming routine practice in several major funding bodies, such as the NSF, the National Institutes of Health and the European Union's Framework Program 7 [83,84]. It would not take long to make such a requirement mandatory by the relevant agencies across the world, which would be good for data publishing. Institutional commitment and mandatory statements by funding agencies and scholarly journals are essential. Data papers can be seen as a step towards peer review and fitness-for-use review of data resources. Data management, especially metadata and data discovery, should be woven into every course in science [28], including, for example, the concepts of big science, small science and incidental science [41]. Data papers also give an opportunity to credit and cite not only academics, but also those who collect and manage data. We are convinced by Rees' [75] prediction that the data paper genre will prove itself useful and will be expanded and enriched so that it takes on the role of filling all gaps in the data reuse pipeline. Creative Commons recommends [75] that granting agencies and tenure review boards see data paper as a legitimate and obligatory activity and that publishers of data papers should make it obligatory that the data resource itself is archived or published in one or more data repositories or network or information system.

## Conclusions

The data paper as an incentive mechanism would achieve increased data discovery and increased accreditation, both of which are desirable to data publishers. It can accelerate the publishing and discovery of biodiversity data resources, helping to justify public investment in biodiversity science. Although it seems straightforward to implement from a technical or infrastructural point of view, it calls for cultural and attitude change on the part of scholarly publishers, scientific societies, funding agencies, data publishers and individual scientists. In our opinion, mainstreaming of data papers would be a step toward elevating data publishing to the level of scholarly publishing and is expected to lead to a significant increase in the efficiency of biodiversity science.

## Competing interests

The authors declare that they have no competing interests.
